# Empagliflozin and colchicine in patients with reduced left ventricular ejection fraction following ST-elevation myocardial infarction undergoing primary percutaneous coronary intervention: a study protocol for a randomized, double-blinded, three-arm parallel-group, controlled trial

**DOI:** 10.1186/s13063-023-07682-6

**Published:** 2023-10-06

**Authors:** Sajad Khiali, Mohammadreza Taban-Sadeghi, Parvin Sarbakhsh, Naser Khezerlouy-Aghdam, Hossein Namdar, Rezvanieh Salehi, Afra Rezagholizadeh, Taher Entezari-Maleki

**Affiliations:** 1 Department of Clinical Pharmacy, Faculty of Pharmacy, Tabriz University of Medical Sciences, Tabriz, Iran; 2 Cardiovascular Research Center, Tabriz University of Medical Sciences, Tabriz, Iran; 3 Department of Statistics and Epidemiology, Faculty of Public Health, Tabriz University of Medical Sciences, Tabriz, Iran

**Keywords:** Empagliflozin, Colchicine, Myocardial infarction, Heart failure, LVSD, Clinical trial

## Abstract

**Background:**

Patients with acute myocardial infarction are at greater risk for chronic heart failure and mortality. Currently, there is limited evidence supporting the beneficial effects of sodium-glucose cotransporter-2 inhibitors on cardiovascular outcomes in non-diabetic patients with reduced left ventricular ejection fraction following acute myocardial infarction. Furthermore, the clinical effects of the combination of standard-dose sodium-glucose cotransporter-2 inhibitors with colchicine and high-dose sodium-glucose cotransporter-2 inhibitors in this setting have not been evaluated yet.

**Methods:**

A prospective, double-blinded, parallel-group, placebo control randomized trial will be carried out at Shahid Madani Heart Center, the largest teaching referral hospital for cardiovascular diseases, affiliated with Tabriz University of Medical Sciences. A total of 105 patients with reduced left ventricular ejection fraction (≤ 40%) following the first episode of ST-elevation myocardial infarction undergoing primary percutaneous coronary intervention with stent insertion will be randomized 1:1:1 to receive empagliflozin 10 mg daily, a combination of empagliflozin 10 mg daily and colchicine 0.5 mg twice daily, or empagliflozin 25 mg daily for 12 weeks. The primary outcomes are changes in the New York Heart Association functional classification and high-sensitivity C-reactive protein from the randomization through week 4 and week 12.

**Discussion:**

The present study will be the first trial to evaluate the efficacy and safety of early treatment with the combination of standard-dose empagliflozin and colchicine as well as high-dose empagliflozin in non-diabetic patients with reduced left ventricular ejection fraction following ST-elevation myocardial infarction. The results of this research will represent a significant step forward in the treatment of patients with acute myocardial infarction.

**Trial registration:**

Clinical trial ID: IRCT20111206008307N39. Registration date: 27 October 2022.

**Graphical Abstract:**

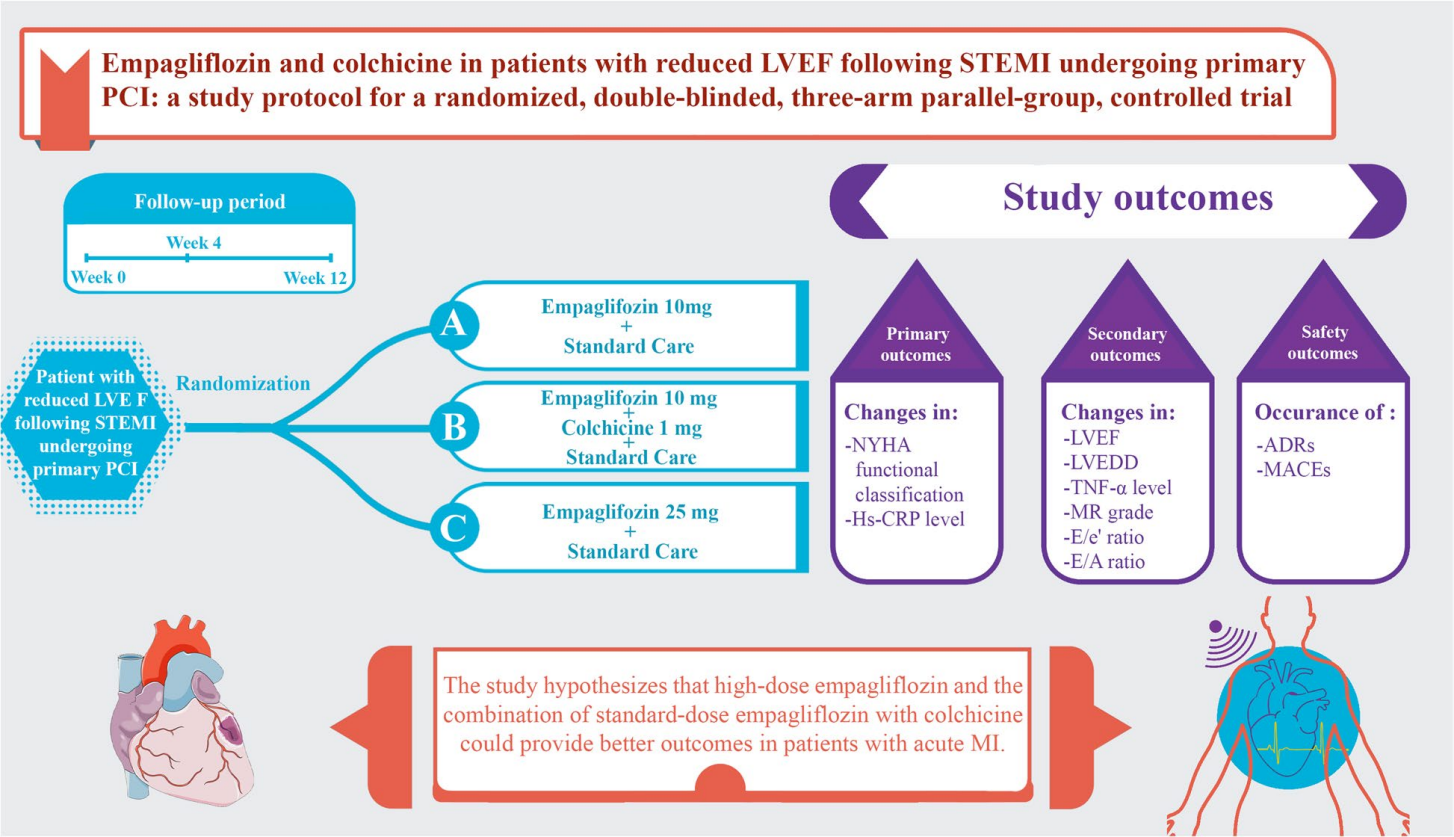

**Supplementary Information:**

The online version contains supplementary material available at 10.1186/s13063-023-07682-6.

## Background

The prevalence of heart failure (HF) increases dramatically due to the aging of the population in most countries and the novel treatments extending the longevity of patients suffering from cardiovascular diseases (CVDs). According to the National Health and Nutrition Examination Survey (NHANES) data, between 2013 and 2016, the estimated prevalence of HF in the USA was approximately 6.2 million. Recently, the global prevalence of HF has been estimated to be over 23 million [1–3]. Left ventricular ejection fraction (LVEF) has been considered a key predictor of cardiovascular mortality and morbidity in HF patients. HF with reduced EF (HFrEF) is defined as HF with LVEF ≤ 40% and accounts for half of all reported patients with HF in the USA [1–4].

It has been estimated that myocardial infarction (MI) affects approximately 605,000 individuals annually [5]. Approximately 40% of patients develop left ventricular systolic dysfunction (LVSD) with or without HF signs following an acute MI episode. It has been shown that a 5% decrease in LVEF during MI hospitalization is associated with a 12–18% increase in the risk of HF development [6].

Sodium-glucose cotransporter-2 (SGLT2) inhibitors have created a relatively novel class of antidiabetic medicines with significant beneficial cardiovascular effects by reducing inflammation, oxidative stress, fibrosis, and sympathetic nervous system activation. Besides, SGLT2 inhibitors could improve mitochondrial function and myocardial competence through modification of myocardial signal transduction by inhibiting the Na + /H + exchanger [7].

There is a growing body of evidence regarding the significant effects of SGLT2 inhibitors on the risk of hospitalization or death due to HF in patients who are chronically suffering from HF. A comprehensive meta-analysis of five randomized controlled trials including 12,251 patients showed that SGLT2 inhibitors could decrease the risk of cardiovascular mortality and morbidity in patients with HF regardless of EF [8]. Accordingly, based on the results of the EMPagliflozin outcomE tRial in patients with chrOnic heaRt failure with Reserved ejection fraction (EMPEROR-Reduced) phase III and EMPagliflozin outcomE tRial in patients with chrOnic heaRt failure with Preserved ejection fraction (EMPEROR-Preserved) phase III trials, the United States Food and Drug Administration (FDA) has approved empagliflozin 10 mg administration for HF regardless of LVEF [9, 10]. Furthermore, the recent guideline regulated by the American Heart Association (AHA), the American College of Cardiology (ACC), and the Heart Failure Society of America (HFSA) has recommended the administration of SGLT2 inhibitors in patients with HFrEF, HF with mildly reduced EF (HFmrEF), or HF with preserved ejection fraction (HFpEF) [4].

The clinical safety and efficacy of the SGLT2 inhibitors have also been evaluated in acute stages of HF. A multinational randomized trial of 530 patients with a primary diagnosis of acute de novo or decompensated HF showed that administration of 10 mg empagliflozin within a median of 72 days from hospitalization could lead to significant clinical benefits [11]. Recently, the EMpagliflozin in patients with acute MYocardial infarction (EMMY) multicenter, double-blind trial showed that administration of 10 mg empagliflozin within 72 h after percutaneous coronary intervention (PCI) in patients with acute MI is associated with a significant reduction in N-terminal prohormone of brain natriuretic peptide (NT-proBNP) levels, mean early diastolic mitral inflow velocity to early diastolic mitral annulus velocity ratio (*E*/*e*′), and left-ventricular end-systolic and end-diastolic volumes, as well as a significant increase in the absolute LVEF over 26 weeks [12].

Colchicine is an ancient plant-derived medicine with well-documented anti-inflammatory properties. Currently, the therapeutic use of colchicine has been extended beyond gout and familial Mediterranean fever (FMF) to CVDs. The broad anti-inflammatory effects of colchicine in CVDs are mediated by several inhibitory mechanisms throughout the inflammation pathway, including migration, adhesion, and activation of neutrophils, the release of matrix metalloproteinases (MMP), defensins, and elastin by neutrophils, nucleotide-binding domain, leucine-rich–containing family, pyrin domain–containing-3 (NLRP3) inflammasome activation, and consequently C-reactive protein (CRP) stimulation; the proliferation of the smooth muscles; and vascular stenosis [13]. In a randomized, double-blind trial on 4745 patients, Tardif et al. showed that administration of 0.5 mg/day colchicine within 30 days following MI is associated with a significantly lower risk of ischemic cardiovascular events [14].

Given the above evidence, we designed the Empagliflozin and COlchicine in patients with MI (ECO-MI) trial to compare the potential beneficial effects of standard-dose empagliflozin, the combination of standard-dose empagliflozin and colchicine, and high-dose empagliflozin in patients with reduced LVEF (≤ 40%) following ST-elevation MI (STEMI) undergoing primary PCI and stent insertion. The ECO-MI is a randomized (1:1:1 allocation ratio), placebo-controlled, double-blinded, single-center superiority trial including three parallel groups.

## Methods and design

### Study design and setting

**Fig. 1:**
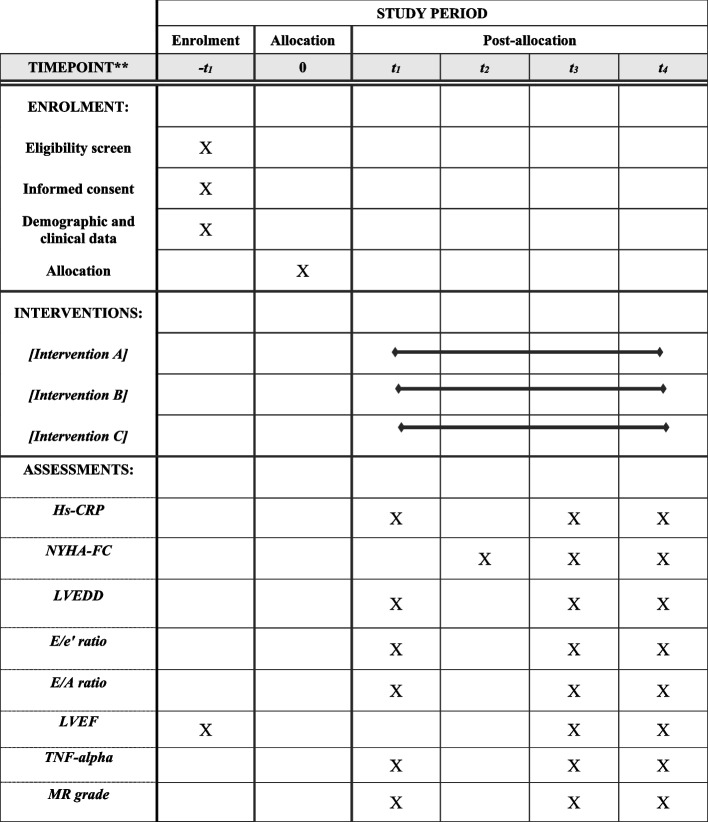
ECO-MI study protocol diagram. Intervention **A**: empagliflozin 10 mg with placebo; intervention **B**: empagliflozin 10 mg with colchicine; intervention C: empagliflozin 25 mg with placebo. − *t*_1_, 3 days pre-allocation; *t*_0_, allocation; *t*_1_, hospital discharge; *t*_2_, 4 weeks after allocation; *t*_3_, 12 weeks after allocation; Hs-CRP, high-sensitivity C-reactive protein; NYHA-FC, New York Heart Association (NYHA) Functional Classification; LVEDD, left ventricular end-diastolic diameter; *E*/*e*′ ratio, early diastolic mitral inflow velocity to early diastolic mitral annulus velocity ratio; E/A ratio, peak early to late diastolic filling velocity ratio; LVEF, left ventricular ejection fraction; TNF alpha, tumor necrosis factor alpha; MR, mitral regurgitation

ECO-MI is a prospective, double-blinded, parallel-group, controlled, randomized trial of hospitalized patients with reduced LVEF (≤ 40%) following the first episode of STEMI undergoing primary PCI with stent insertion. Patients will be recruited for the study at Shahid Madani Heart Center, the largest teaching referral hospital for CVDs affiliated with Tabriz University of Medical Sciences, Tabriz, Iran. The ECO-MI protocol follows the Standard Protocol Items Recommendations for Interventional Trials (SPIRIT) guidelines (Fig. 1 and Additional file 1) [15]. The recruitment data will be illustrated using the CONSORT flow diagram [16].

### Eligibility criteria

During the screening and study period, a cardiologist (MT) will assess the patients for eligibility and collect demographic and medical history. Additionally, a urine pregnancy test in women with childbearing potential will be obtained. The inclusion criteria are as follows: [1] first episode of STEMI undergoing primary PCI within 72 h, [2] reduced left ventricular ejection fraction (LVEF ≤ 40%), [3] 18 to 80 years of age, and [4] consenting to the study. The exclusion criteria include [1] history of chronic HF or reduced LVEF to equal or less than 40% prior to the MI; [2] diabetes mellitus; [3] current evidence of cardiogenic shock; [4] hemodynamic instability as defined by intravenous administration of catecholamine, calcium sensitizers, or phosphodiesterase inhibitors; [5] hypoglycemia defined as a blood glucose level of less than 70 mg/dL; [6] blood pH less than 7.32; [7] currently being treated with SGLT-2 inhibitors or colchicine or having received SGLT-2 inhibitors or colchicine within 4 weeks before the screening visit; [8] estimated glomerular filtration rate (eGFR) less than 30 mL/min/1.73 based on the chronic kidney disease epidemiology collaboration (CKD-EPI) formula [17]; [9] end-stage renal disease; [10] receiving dialysis; [11] severe hepatic disease with Child–Pugh score of equal or more that 7 (grade B or C) [18, 19]; [12] acute symptomatic urinary tract infection or genital infection; [13] operated within the recent 3 years or planned coronary artery bypass grafting (CABG); [14] inflammatory bowel disease or chronic diarrhea; [15] history of cancer within the previous 3 years, [16] autoinflammatory diseases; [17] current or planned long-term systemic glucocorticoid therapy; [18] neuromuscular diseases or a non-transient creatine kinase level greater than three times the upper limit of the normal range (except for the infarction-based cases), [19] clinically significant non-transient blood dyscrasia; [20] drug or alcohol abuse; [21] inability to be assessed by the New York Heart Association (NYHA) functional class; [22] hypersensitivity to empagliflozin, colchicine, or other pharmaceutical compounds in used formulations; [23] pregnancy; [24] females of childbearing potential without adequate contraceptive methods (i.e., sterilization, intrauterine device, vasectomized partner; or medical history of hysterectomy); and [25] lactation.

### Interventions

Patients will be randomized 1:1:1 to receive empagliflozin 10 mg daily (group A), a combination of empagliflozin 10 mg daily and colchicine 0.5 mg twice daily (group B), or empagliflozin 25 mg daily (group C) for 12 weeks. Additionally, patients in groups A and C will receive a placebo instead of colchicine. All patients will be treated according to the 2022 AHA/ACC/HFSA guidelines for the management of HF [4]. Besides, all patients may be administered appropriate medications for concomitant comorbidities. All patients will be asked not to take any medication before consulting with a cardiologist (HN).

All patients will be guided to report any clinical presentation of drug-related adverse reactions to the researchers as soon as possible during the hospitalization period, the fourth week, the twelfth week, and the weekly telehealth visits. For empagliflozin, adverse reactions such as acute kidney injury, hypersensitivity, fluid and electrolyte abnormalities, infection, and ketoacidosis will be explained. Similarly, adverse reactions such as gastrointestinal, nerve system, hepatic, hematologic, neuromuscular, and skeletal events will be described in the case of colchicine use. The management of reported adverse reactions will be done by HN. All patients will be asked to use empagliflozin once daily in the morning with or without food, take colchicine in two divided doses with or without food, maintain adequate fluid intake, take the missed dose as soon as possible, and skip the missed dose if it is close to the time for the next dose instead of taking two doses at the same time.

### Patient adherence

The study protocol will be explained by the researchers in detail by SK. A medication schedule template will be given to each patient to increase their adherence to the study. Participants will be asked to contact the researchers if they have any questions or health concerns during the study period. They will receive their medication packages at the baseline and the fourth week. In each visit, all patients will be asked to return any unused medications to assess their level of compliance. The remaining medications will be gathered in week 4 and week 12. If less than 90% of the medications have been used in each visit, the person would be considered non-adherent. More precisely, patients’ adherence will be reassessed using the Eight-Item Morisky Medication Adherence Scale (MMAS-8). Patients with MMAS-8 scores of less than 6 will be considered non-adherent [20]. For data analysis, the intention-to-treat (ITT) analysis approach with the multiple imputation method will be implemented [21, 22].

### Primary outcomes

The primary outcomes include changes in the NYHA functional classification and high-sensitivity C-reactive protein (hs-CRP) levels since the randomization through weeks 4 and 12.

### Secondary outcomes

The secondary objectives consist of changes in LVEF, tumor necrosis factor alpha (TNF-α), and echocardiographic parameters, including left ventricular end-diastolic dimension (LVEDD), mitral regurgitation (MR) grade, *E*/*e*′ ratio, and peak early to late diastolic filling velocity ratio (*E*/*A*) from the time of randomization to week 4 and week 12.

### Safety events

The occurrence of significant adverse drug reactions (ADRs), including acute kidney injury, bone fracture, hypotension/volume depletion, ketoacidosis, lower limb amputations, genitourinary infections, hypoglycemia, and gastrointestinal events will be monitored. Finally, the incidence of major adverse cardiac events (MACEs) including MI, revascularization, stroke, cardiovascular mortality, and hospitalization because of HF will be evaluated during the follow-up period of 12 weeks.

### Sample size

The sample size was calculated based on the primary outcomes of the trial. The sample size for the NYHA functional class, presuming a significance level of 0.05 and a power of 80%, according to the expected effect size of 40% from the EMPEROR-Reduced trial on the effects of empagliflozin on the NYHA functional class based on proportions comparison sample size formula was calculated 23 cases for each group and totally 69 patients [23]. According to the information from Fiolet et al.’s study on the effect of colchicine on hs-CRP and estimating 3.5 mg/dL as the standard deviation, the expected effect size *f* for colchicine was calculated to be 0.33. Thereafter, presuming a significance level of 0.05 and a power of 80% as well as taking into account the analysis of variance sample size formula group, the required sample size for this outcome was calculated to be 93 cases in total with 31 patients in each group [24]. So, the maximum value of these sample sizes (93 cases) will be considered to conduct the study. The calculation of sample size was conducted using the G*power software [25]. The mentioned sample size would increase to at least 105 patients in practice, considering the attrition rate of 10%. The actual code used in G*power to compute the sample size is provided in supplementary material.

### Recruitment

In the ECO-MI single-center trial, medical reports of patients will be thoroughly evaluated for subject identification and recruitment.

### Randomization and blinding

Randomization will be carried out using online random allocation by blocked randomization method with random block sizes of 6 and 9 [26]. The method of sequentially numbered, opaque sealed envelopes (SNOSE) will be used for allocation concealment [27]. A statistics specialist (PS) will generate the allocation sequence, MT will enroll participants, and SK will assign participants to interventions. Once the patient is considered eligible for the study, SK will open a sealed opaque envelope with ordered serial numbers to assign participants to interventions. During the study, all investigators involved directly to the study and patients will be blind throughout the procedure and will not know which treatment has been administered. The placebo is identical to colchicine in every aspect, including color, appearance, smell, and taste. HN who is not involved in the recruitment process, data collection, and data analysis will be unblinded in order to manage, collect, assess, and report solicited and spontaneously reported adverse events and other unintended effects of trial interventions.

### Data management

Each patient will be given a secret code to keep their information confidential. The informed consent patients’ codes, personal information, demographic and clinical data, and visit dates will be recorded by the researchers in the data reporting form of each patient. Demographic and clinical data include age, gender, ethnicity, weight, height, body mass index, complete blood count, serum creatinine, lipid profile, liver function test, serum albumin, partial thromboplastin time, prothrombin time, hemoglobin A1C, blood sugar, thyroid-stimulating hormone, lactate dehydrogenase, creatine kinase, creatine kinase MB, cardiac troponin I, electrocardiogram findings, eGFR, Child–Pugh score, familiar history of CVD, coronary angiography vessel status, drug history, past medical history, serum levels of hs-CRP, TNF-α, MDA, and TAC, echocardiographic findings, NYHA functional class, ADRs, and MACEs. All data will be reviewed and kept by the project coordinator, who will be responsible for data processing and checking the completeness and accuracy of data input. Any unexpected or out-of-range data values will be double-checked by the researchers before data analyses.

### Outcome measurements

Baseline echocardiography and blood sampling will be carried out before the administration of the first dose of investigational medications. The demographic data and clinical information of patients will be documented in data-collecting forms by MT. The arranged face-to-face visits, echocardiography, and blood sampling will be performed during the fourth and twelfth weeks. Assessment of the NYHA function class was carried out in the arranged face-to-face visits by MT. Patients will be assessed regarding ADRs and MACEs during the study period using weekly telehealth and arranged face-to-face visits.

Echocardiography will be carried out in accordance with the current guidelines of the American Society of Echocardiography (ASE) and the European Association of Cardiovascular Imaging (EACVI) [24]. The Philips Epiq 7C cardiology ultrasound machine (Los Angeles, CA, USA) will be used to perform two-dimensional, M-mode, spectral Doppler (pulsed and continuous wave), color flow Doppler, and tissue Doppler. Echocardiography will be conducted by MT. All data will be stored in digital imaging and communications in medicine (DICOM) format [28].

Blood samples for immunoassay will be collected from all patients at the baseline, week 4, and week 12 following the allocation. All blood samples will be centrifuged at the speed of 1500 RPM for 20 min. Then, the serum will be collected into two separate micro-tubes and stored in a freezer at a temperature of − 70 °C until the analysis. The level of serum CRP will be measured using hs-CRP immunoassay test kits (Aptec Diagnostics, 9100 Sint-Niklaas, Belgium; 9100 Sint-Niklaas, Belgium). The measurement range is 0 to 14 mg/dL with a detection limit of 0.013 mg/dL. The risk of cardiovascular diseases will be determined based on the reference values of the kits which are defined as follows: less than 0.10 mg/dL as low-risk, 0.10 to 0.30 mg/dL as intermediate-risk, and greater than 0.30 mg/dL as high-risk. Moreover, the level of serum TNF-α will be quantified based on the enzyme-linked immunosorbent assay (ELISA) kit (Bioassay Technology Laboratory Cat. No.Eo.E0082Hu, Shanghai, China). The assay range is 3 to 900 ng/L with a detection limit of 1.52 ng/L. One micro-tube of sampling will be stored in a freezer at a temperature of − 70 °C for the measurement of empagliflozin serum concentration, total antioxidant capacity, and malondialdehyde level.

### Statistical analysis

The data will be analyzed using SPSS version 22 (SPSS Inc., Chicago, IL, USA, 2013). According to the data distribution, which will be checked by graphical and numerical methods, the description of the data will be done with the appropriate central tendency and dispersion indices. The within-group and between-group comparisons will be done using the analysis of variance (ANOVA) tests. The final effect of the interventions will be evaluated by assessing the interaction effect of the group and time in the repeated measures model considering the group and time as the between-subject and within-subject effects, respectively. To adjust the effect of confounders and important covariates, multivariable repeated measures will be used.

### Trial steering and data monitoring committees

Considering the single-center nature of the trial, the research team will be responsible for steering or monitoring the study. Patients will be under constant monitoring, and any adverse events would be recorded by the investigators. Additionally, monthly meetings managed by the head investigator (TE) are held to maintain constant communication among the researchers and audit the trial conduct. As the principal investigator, TE is responsible for the design and conduct of the trial, organizing committee meetings, and publication of study results and reports. Monthly trial steering committee meetings managed by the head investigator will be held to maintain constant communication among the researchers and audit the trial conduct. Day-to-day trial support will be provided by a core group, including the principal investigator, cardiologists, a biostatistician, and a clinical pharmacist, responsible for the trial progression, adherence to the protocol, patient safety, and consideration of new information relevant to the research question. A data monitoring committee is not required during the trial period since there is a low risk of adverse events. Tabriz University of Medical Sciences will provide organizational support. An independent clinical event committee consisting of cardiologists, clinical pharmacists, and a biostatistician will provide an independent expert review of critical endpoints reported by investigators to adjudicate clinical events and determine whether the endpoints meet the protocol-specific criteria periodically. All serious adverse events, complications, and harms (in case of any) will be reported to the Research Ethics Committee of Tabriz University of Medical Sciences and documented.

### Patient and public involvement

There is no direct patient or public involvement in the study. However, to motivate the active involvement of patients throughout the research process, participants will be monitored regularly. Besides, the researchers will explain the study question, the benefits and harms of the protocol, and the beneficial impact of the investigation on public health in detail to the participants. Offering free medical services that are not obligatory in each visit will be performed, and eventually, the participants will be encouraged to contact the investigators at any time for consultations and medical advice.

## Discussion

This ongoing study is a prospective, double-blinded, three-arm, parallel-group, placebo-controlled trial designed to evaluate the efficacy and safety of standard-dose empagliflozin, high-dose empagliflozin, and the combination of standard-dose empagliflozin and colchicine in patients with reduced LVEF following first episode of STEMI undergoing primary PCI with stent insertion. The primary objective of this randomized controlled trial (RCT) is to evaluate the changes in hs-CRP and the NYHA functional classification during the study period of 12 weeks. The study hypothesizes that high-dose empagliflozin and the combination of standard-dose empagliflozin with colchicine could provide better outcomes in patients with reduced LVEF following STEMI undergoing primary PCI.

### HF following MI

It has been estimated that HF incidences following acute MI range from 14 to 36% [29]. Among patients with MI, there is a strong correlation between the degree of HF and death [5, 30]. HF development following MI could occur at the onset of MI, during the hospitalization for MI, and after discharge with dissimilar pathophysiology, clinical characteristics, and clinical outcomes. Mechanistically, myocardial necrosis, myocardial stunning, and mechanical complications have been shown to cause HF development during hospitalization. Inflammatory responses secondary to cardiomyocyte structural changes and cell death have been shown to play a fundamental role in the process of HF development. In addition, mounting evidence has supported the central role of the renin–angiotensin–aldosterone system (RAAS) and chronic neurohumoral changes in the pathophysiology of ventricular remodeling and HF development after hospital discharge for acute MI [30, 31]. Targeting these mechanisms could underpin the development of novel treatments to decrease HF risk following MI. Identifying patients at higher risk for HF (stage A) or pre-HF (stage B) could provide an opportunity to initiate appropriate medications to prevent or delay the progression of the disease to stages C and D.

### SGLT2 inhibitors in HF

A mounting body of evidence strongly supports the beneficial effects of SGLT2 inhibitors in the prevention of hospitalizations due to HF in type 2 diabetic patients who are suffering from or are at risk for CVD [32–35]. The 2022 AHA/ACC/HFSA guideline for the management of HF recommended the use of SGLT2 inhibitors in patients with type 2 diabetes mellitus and stage A or stage B of HF [4].

The beneficial effects of SGLT2 inhibitors are independent of their glucose-lowering properties and have been suggested to be by the reduction in cardiac preload and afterload, modification of cardiac metabolism, decrease in arterial stiffness, and interaction with the Na + /H + exchanger. According to the promising findings of the EMPEROR-Reduced, the EMPEROR-Preserved, and the EMPULSE trials, empagliflozin significantly reduces the risk of cardiovascular death or hospitalization in a wide spectrum of patients with HF, irrespective of the presence of type 2 diabetes mellitus [11, 24, 36]. The 2022 AHA/ACC/HFSA guideline also has recommended the use of SGLT2 inhibitors in patients with chronic symptomatic HF with or without type 2 diabetes to reduce CVD mortality and HF hospitalization [4]. The EMPULSE trial supported the initiation of empagliflozin as a part of the usual care in patients with acute HF to improve all-cause mortality, HF events, and quality of life without safety concerns [11]. Consequently, one important missing piece of the puzzle would be the efficacy and safety of SGLT2 inhibitors in non-diabetic patients with stage A or stage B of HF who are at high risk for HF development or progression.

Patients with LVSD following an episode of acute STEMI are at greater risk of HF [37]. In the ECO-MI trial, non-diabetic patients with moderate to severe LVSD following STEMI will be studied. In case of hypothesis approval, this trial will open a new insight into the treatment of patients with acute MI suffering LVSD.

### SGLT2 inhibitors in acute MI

Pharmacologically, SGLT2 inhibitors could be beneficial in MI by inhibiting the inflammatory pathways, modification of myocardial signal transduction through inhibition of Na + /H + exchanger, and metabolic effects [7]. Irrespective of diabetes, empagliflozin could improve cardiac remodeling parameters and ameliorate fibrosis and hypertrophy by overexpression of the guanosine triphosphate cyclohydrolase I (GTPCH 1) enzyme [38]. The cardiovascular benefits of SGLT2 inhibitors onset within weeks, which is the golden time to prevent the pathophysiological pathways following MI [36, 39]. This issue further highlights the clinical application of SGLT2 inhibitors in post-MI patients.

The EMMY trial represented a significant step forward for SGLT2 inhibitors in patients with acute MI. In the EMMY trial, patients were included regardless of their diabetes status, which could result in difficulty in the interpretation of the data. Another limitation of the EMMY trial may be that only 18% of the patients were female [12]. In the EMMY trial, the efficacy and safety of empagliflozin 10 mg in post-MI patients were revealed.

Given the significant prognostic implication of LV dysfunction following acute MI in HF mortality and sudden cardiac death, the ECO-MI trial will include non-diabetic patients with new moderate to severe LV dysfunction (LVEF < 40%) following acute STEMI [40]. Furthermore, the ongoing DAPAgliflozin on the prognosis of patients with acute Myocardial Infarction (DAPA-MI) and EMPAgliflozin on hospitalization for heart failure and mortality in patients with aCuTe myocardial infarction (EMPACT-MI) trials will shed the light on the efficacy and safety of SGLT-2 inhibitors in patients with new cardiac dysfunction after MI [41].

Recently, the efficacy and safety of standard and high doses of empagliflozin in patients with chronic HF have been demonstrated. According to Hao et al., compared to empagliflozin 10 mg, empagliflozin 25 mg could further decrease the risk of HF hospitalization in chronic HFrEF patients with similar safety profiles [42]. In the present trial, we will compare the potential effects of two different doses (10 and 25 mg) of empagliflozin in patients with recent STEMI and at high risk for HF development.

The NYHA function classification has been used clinically for more than 100 years as a foundational tool for HF classification and determining patients’ eligibility to receive certain treatments [43]. In addition, there is a substantial correlation between higher NYHA functional class and poor HF outcomes [30]. According to the 2022 AHA/ACC/HFSA guideline for the management of HF, the NYHA functional classification is an independent predictor of mortality [4]. In the EMPEROR-Reduced trial, patients who received empagliflozin 10 mg once daily experienced 20 to 40% higher odds of NYHA functional class improvement and 20 to 40% lower odds of NYHA functional class worsening within the follow-up period of 52 weeks. These beneficial findings were observed from the first visit which was conducted 4 weeks after randomization [23]. Similarly, in the EMPEROR-Preserved trial, patients in the empagliflozin group had 20 to 50% higher odds of experiencing an improvement in NYHA functional class in each visit from week 12 to week 148 [36]. The ECO-MI trial will evaluate the changes in the NYHA function class from baseline to week 4 and week 12.

### Colchicine in acute MI

Today, colchicine has also emerged with promising results in the management of cardiovascular diseases. It has been hypothesized that colchicine, a potent anti-inflammatory agent, could have beneficial effects in patients with acute MI due to its anti-atherosclerotic, anti-inflammatory, anti-arrhythmic, and anti-fibrotic effects, which has been supported by preclinical studies [13, 44]. Accordingly, multiple RCTs aimed to evaluate the efficacy and safety of colchicine in patients with MI. A double-blind, multicenter RCT was carried out to evaluate the potential clinical benefits of colchicine in patients with STEMI undergoing primary PCI. The associated data analysis revealed that oral administration of colchicine with a loading dose of 2 mg at the time of reperfusion followed by 0.5 mg twice daily for 5 days could not significantly improve LV end-diastolic volume (*P* = 0.49) and infarct size (*P* = 0.87) [44]. Although evaluating the short-term use of colchicine in the acute phase of ischemia following MI was not promising, Tardif et al. in a randomized, double-blind trial showed the beneficial effects of long-term colchicine administration in 4745 patients with a recent MI. According to Tardif et al., the primary endpoint, including death from cardiovascular causes, resuscitated cardiac arrest, myocardial infarction, stroke, and urgent hospitalization due to angina leading to coronary revascularization, was significantly lower in patients who received colchicine 0.5 mg daily compared with the placebo group during a median follow-up of 22.6 months (5.5% vs. 7.1%; hazard ratio, 0.77; 95% confidence interval [CI], 0.61 to 0.96; *P* = 0.02) [14].

Moreover, according to a systematic review and meta-analyses of six RCTs involving 6005 patients who recently had experienced acute MI, colchicine did not lead to a significant decrease in cardiovascular mortality (risk ratio [RR], 0.91; 95% CI, 0.52–1.61; *P* = 0.64), recurrent MI (RR, 0.87; 95% CI, 0.62–1.22; *P* = 0.28), all-cause mortality (RR, 1.06; 95% CI, 0.61–1.85; *P* = 0.78), stroke (RR, 0.28; 95% CI, 0.07–1.09; *P* = 0.05), or urgent coronary revascularization (RR, 0.46; 95% CI, 0.02–8.89; *P* = 0.19). Similarly, no significant difference was observed regarding total adverse events (RR, 0.97; 95% CI, 0.89–1.07; *P* = 0.34) or specific gastrointestinal adverse events (RR, 2.49; 95% CI, 0.48–12.99; *P* = 0.20) [45].

Despite the relatively long half-life of colchicine (27–31 h), the 2015 guidelines of the European Society of Cardiology (ESC) for the diagnosis and management of pericardial diseases have recommended against the loading dose administration of colchicine in pericarditis due to the lower compliance of the patients [46]. In the ECO-MI trial, a group of patients with acute STEMI undergoing primary PCI with stent insertion will receive the combination of colchicine 0.5 mg twice daily (without a leading dose) and empagliflozin 10 mg for 3 months.

According to a growing body of literature, there is a correlation between the serum level of hs-CRP and the risk of MACEs in patients with MI [47, 48]. Carrero et al. in a healthcare‐based study showed that patients with hs-CRP ≥ 2 mg/L were at a greater risk of MACEs (*n* = 3900; adjusted hazard ratio, 1.28; 95% CI, 1.18–1.38) and mortality (*n* = 4138; adjusted hazard ratio, 1.42; 95% CI, 1.31–1.53) within 30 days following MI. Notably, the correlation between the hs-CRP level and the incidence of MACEs and mortality was linear when 1 < hs-CRP < 5 mg/L [47]. A randomized, double-blind RCT on 237 patients indicated that colchicine 0.5 mg daily could not significantly decrease absolute levels of CRP 1 month after an acute MI [7]. In the study carried out by Tardif et al., adjusted geometric mean percentage changes of hs-CRP serum levels in 207 patients within 6 months were − 70.0% and − 66.6% for the colchicine and placebo groups, respectively [14].

Furthermore, a randomized, double-blind, placebo-controlled trial on 150 patients with non-STEMI indicated that administration of colchicine for 30 days could lead to lower hs-CRP levels compared with placebo (*P* < 0.001). It is worth mentioning that hs-CRP levels did not decrease to less than 2 mg/L in both groups [49].

In the present trial, we will compare the effect of empagliflozin 10 mg daily, empagliflozin 10 mg daily plus colchicine 0.5 mg twice daily, and empagliflozin 25 mg daily on hs-CRP serum levels in patients with LV dysfunction following STEMI undergoing primary PCI with stent insertion during a follow-up period of 12 weeks.

### Strengths and limitations of the study

The ECO-MI will be the first trial to present data on early treatment with SGLT2 inhibitors in non-diabetic patients following MI with reduced EF. The ECO-MI trial is the first trial designed to evaluate the effects of a high-dose SGLT2 inhibitor and a combination of colchicine with a standard-dose SGLT2 inhibitor in patients following MI. The primary outcomes of the trial are of significant importance in indirect mortality prediction and the development of HF in post-MI patients.

As a nature of any research, ECO-MI may have some limitations. While the sample size was calculated for the two primary outcomes, ECO-MI does not seem to be powerful enough to investigate the echocardiographic parameters, hospitalization for HF, cardiovascular mortality, and all-cause mortality outcomes. The study duration is limited in this study. The predicted positive primary outcome in the ECO-MI will not necessarily appear to translate into mortality benefits. Finally, although colchicine metabolism is influenced by genetic variations, genetic data will not be collected to assess predisposition to colchicine resistance; however, the RCT nature of the study could effectively neutralize this effect [50].

### Supplementary Information

**Additional file 1. **SPIRIT 2013 Checklist: Recommended items to address in a clinical trial protocol and related documents*.

## Data Availability

The protocol is part of SK’s Ph.D. thesis. By the end of the trial, all primary and secondary outcome data will be reported and published. The participant-level data will be available from the corresponding author upon reasonable request.

## Abbreviations

ACC: American College of Cardiology
ADR: Adverse drug reaction
AHA: American Heart Association
ANOVA: Analysis of variance
ASE: American Society of Echocardiography
CABG: Coronary artery bypass grafting
CI: Confidence interval
CKD-EPI: Chronic Kidney Disease EPIdemiology Collaboration
CONSORT: Consolidated Standards Of Reporting Trials
CRP: C-reactive protein
CVD: Cardiovascular disease
DAPA-MI (study): DAPAgliflozin on the prognosis of patients with acute Myocardial Infarction
DICOM: Digital Imaging and COmmunications in Medicine
EACVI: European Association of CardioVascular Imaging
EF: Ejection fraction
EGFR: Estimated glomerular filtration rate
ELISA: Enzyme-linked immunosorbent assay
EMMY (study): EMpagliflozin in patients with acute MYocardial infarction
EMPACT-MI (study): EMPAgliflozin on hospitalization for heart failure and mortality in patients with aCuTe myocardial infarction
EMPEROR-Preserved (study): EMPagliflozin outcomE tRial in patients with chrOnic heaRt failure with Preserved ejection fraction
EMPEROR-Reduced (study): EMPagliflozin outcomE tRial in patients with chrOnic heaRt failure with Reduced ejection fraction
ESC: European Society of Cardiology
FDA: Food and Drug Administration
FMF: Familial Mediterranean fever
GTPCH1: Guanosine triphosphate cycloHydrolase I
HF: Heart failure
HFmrEF: Heart failure with mildly reduced ejection fraction
HFpEF: Heart failure with preserved ejection fraction
HFrEF: Heart failure with reduced ejection fraction
HFSA: Heart Failure Society of America
Hs-CRP: High-sensitivity C-reactive protein
ITT: Intention-to-treat
LVEDD: Left ventricular end-diastolic dimension
LVEF: Left ventricular ejection fraction
LVSD: Left ventricular systolic dysfunction
MACE: Major adverse cardiac events
MI: Myocardial infarction
MMAS-8: Morisky Medication Adherence Scale
MMP: Matrix metalloproteinases
MR: Mitral regurgitation
NHANES: National Health and Nutrition Examination Survey
NLRP3: Nucleotide-binding domain, leucine-rich–containing family, Pyrin domain–containing-3
NT-proBND: N-terminal prohormone of brain natriuretic peptide
NYHA: New York Heart Association
PCI: Percutaneous coronary intervention
RAAS: Renin-angiotensin aldosterone system
RCT: Randomized controlled trial
RR: Risk ratio
SGLT-2: Sodium-glucose cotransporter-2
SNOSE: Sequentially numbered opaque sealed envelopes
SPIRIT: Standard Protocol Items Recommendations for Interventional Trials
STEMI: ST-elevation myocardial infarction
TNF-α: Tumor necrosis factor-alpha
WHO: World Health Organization
